# Efficacy and challenges of immunotherapy in advanced *EGFR*-mutant NSCLC: a case report

**DOI:** 10.3389/fonc.2026.1846975

**Published:** 2026-06-30

**Authors:** Yue Su, Weigang Dong, Yan Yin, Jianwen Qin

**Affiliations:** Department of Respiratory and Critical Medicine, Tianjin Chest Hospital, Tianjin, China

**Keywords:** EGFR-sensitizing mutation, EGFR-TKI resistance, non-small cell lung cancer (NSCLC), tertiary lymphoid structures (TLS), tumor immune microenvironment (TIME)

## Abstract

Non-small cell lung cancer (NSCLC) harboring EGFR-sensitizing mutations is typically characterized as an immunologically “cold” phenotype, exhibiting limited response to immune checkpoint inhibitors (ICIs). While EGFR tyrosine kinase inhibitors (EGFR-TKIs) represent the standard of care, acquired resistance is inevitable. Herein, we report a case of advanced NSCLC with an EGFR exon 19 deletion (19del). Following progression on multiple lines of TKI therapy and chemotherapy, the patient achieved a progression-free survival (PFS) of 8 months and an overall survival (OS) of 16 months upon receiving a PD-L1 inhibitor-based combination immunotherapy. To elucidate the mechanisms underlying this unexpected clinical benefit, multiplex immunofluorescence (mIF) analysis was performed on serial biopsy specimens obtained pre-immunotherapy (post-osimertinib progression) and post-immunotherapy progression. Results demonstrated that despite low PD-L1 expression in the pre-immunotherapy “responsive” specimen, the tumor immune microenvironment (TIME) exhibited a highly “inflamed” profile, characterized by high-density infiltration of T cells and B cells, as well as the formation of mature tertiary lymphoid structures (TLS). Notably, these TLS structures were virtually absent in the post-progression specimen. These preliminary, single-case observations raise the hypothesis that mature TLS formation following EGFR-TKI resistance may be associated with an immunotherapy-responsive TIME in EGFR-mutation NSCLC – A feature not captured by PD-L1 expression alone. Given the inherent limitations of single-case evidence, these findings are strictly exploratory and hypothesis-generating, and merit further prospective validation in adequately powered cohort studies.

## 1 Introduction

Lung cancer is the leading cause of cancer-related morbidity and mortality worldwide (1). Non-small cell lung cancer (NSCLC) accounts for approximately 85% of cases (2). Sensitizing mutations in the epidermal growth factor receptor gene (EGFR) represent the most prevalent driver mutation subtype, particularly among Asian populations, where the incidence is 30–50% (3). Targeted therapy with EGFR receptor-tyrosine kinase inhibitors (EGFR-TKIs) is the standard first-line treatment for advanced EGFR-mutant NSCLC. The transition from first-generation agents such as gefitinib to the third-generation inhibitor osimertinib has dramatically improved patient prognosis (3). However, the development of acquired resistance is inevitable, rendering subsequent treatment options after TKI treatment failure challenging (3).

Recently, immune checkpoint inhibitors (ICIs) have revolutionized the treatment of NSCLC lacking driver mutations. Conversely, EGFR-mutant NSCLCs are typically immunologically “cold” (4), exhibiting a non-inflamed tumor immune microenvironment (TIME). They have low tumor mutational burden (TMB) and few tumor-infiltrating lymphocytes (TILs) (5). Consequently, the objective response rate to single-agent ICI therapy after TKI failure in patients with EGFR-mutant NSCLC is low, with limited clinical benefits (6, 7).

However, a subset of patients with EGFR-TKI-resistant NSCLC, particularly those who have undergone multiple lines of therapy, may benefit from ICIs, especially in combination regimens (4). This finding implies that the EGFR-TKI treatment course may induce TIME remodeling, potentially creating a new “window of opportunity” for immunotherapy.

The pivotal roles of intratumoral B cells and tertiary lymphoid structures (TLS) in regulating antitumor immunity and predicting ICI efficacy are increasingly recognized (8, 9). TLS are ectopic lymphoid organs that function as local immune “hubs” (8) and their presence, particularly when mature, correlates with favorable ICI outcomes in multiple cancer types (9). In EGFR wild-type tumors, synergistic interaction among T follicular helper (Tfh) cells, B cells, and tissue-resident memory T (Trm) cells are essential for TLS formation and effective anti-PD-1 responses; This synergy is impaired in EGFR-mutation tumors, contributing to primary ICI resistance (5).

Therefore, characterizing TIME evolution following EGFR-TKI resistance, and the dynamic changes in TLS in particular, may help identify patient subgroups most likely to benefit from immunotherapy (3). Herein, we report a patient with advanced EGFR-mutant NSCLC, who achieved a durable response to combination immunotherapy following resistance to multiple lines of targeted therapy. Combined with a literature review, this report aims to explore the feasibility of TIME remodeling after EGFR-TKI resistance and to discuss potential future directions of immunotherapy in this patient population.

## 2 Case presentation

A 69-year-old female was admitted in May 2021, presenting with a 3-month history of intermittent cough and expectoration. Contrast-enhanced chest computed tomography revealed a mass in the right middle lobe, multiple metastatic lesions, and concurrent acute pulmonary embolism (**Figure 1a**). Anticoagulation therapy was initiated immediately. As the patient was intolerant to bronchoscopy, circulating tumor DNA (ctDNA) analysis via Next-Generation Sequencing (NGS) was performed on the peripheral blood, identifying an EGFR exon 19 deletion (p.L747_P753delinsS) with a variant allele frequency (VAF) of 0.95%.

**Figure 1:**
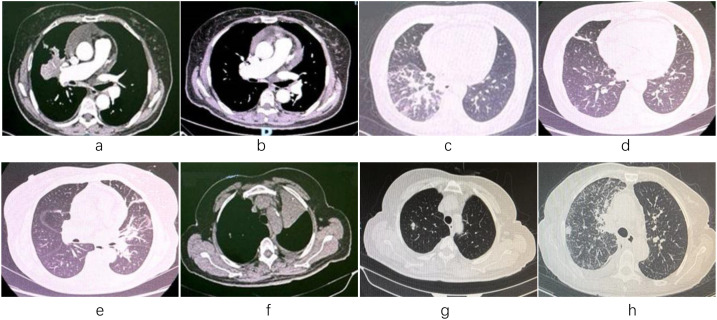
Radiographic evaluation of the patient’s clinical course. **(a)** May 12, 2021: Baseline contrast-enhanced chest CT revealed a tumor in the right middle lobe complicated by right-sided lymphangitis carcinomatosa and partial obstructive pneumonia. Multiple metastatic lymph nodes were observed in the bilateral hila and mediastinum, along with multiple bilateral pulmonary metastases, right-sided pleural effusion, pericardial effusion, and multiple left-sided pulmonary emboli. **(b)** July 2021 (after 2 months of gefitinib therapy): Follow-up chest CT showed marked regression of the right middle lobe lesion, alleviation of lymphangitis carcinomatosa and obstructive pneumonia, and a reduction in the size of multiple bilateral pulmonary micronodules compared to baseline. **(c)** February 18, 2022: Chest CT indicated disease progression, characterized by the emergence of new patchy opacities in the right lower lobe and the enlargement of multiple bilateral pulmonary micronodules. **(d)** During osimertinib treatment: Radiographic improvement of pulmonary lesions was observed. **(e)** June 2023: Chest CT identified new lesions in the lingular segment of the left upper lobe and the basal segment of the left lower lobe. **(f)** Post-chemotherapy: Development of atelectasis in the lingular segment of the left upper lobe was noted following treatment with pemetrexed plus carboplatin and subsequent pemetrexed plus anlotinib. **(g)** Post-immunotherapy (2 cycles): Follow-up chest CT demonstrated significant resolution of the atelectasis in the left upper lobe and lingular segment following treatment with atezolizumab, paclitaxel, and anlotinib. **(h)** December 2024: Chest CT revealed enlargement of patchy opacities in the right middle lobe, progression of multiple bilateral pulmonary nodules, and aggravated thickening of the interlobular septa.

First-line therapy with gefitinib was initiated in May 2021. A partial response (PR) was documented after 2 months (**Figure 1b**), with progressive disease (PD) at 9 months (February 2022; **Figure 1c**). Bronchoscopic biopsy confirmed lung adenocarcinoma staged cT4N3M1 (Stage IV, bilateral lungs, pleura, pericardium, adrenal glands, abdominal lymph nodes; ECOG PS 1). Tissue NGS analysis revealed persistence of the EGFR 19del and emergence of the EGFR T790M mutation.

Second-line Osimertinib was administered; PR was achieved after 2 months and maintained for a PFS of 16 months (**Figures 1d, e**). Disease progression was noted in June 2023. A repeat bronchoscopic biopsy performed on June 28, 2023 (designated the “pre-immunotherapy specimen” [ID: 23-2554]) detected only EGFR 19del by tissue NGS.

Third-line pemetrexed plus carboplatin and fourth-line pemetrexed plus anlotinib both yielded suboptimal responses (**Figure 1f**). Given preserved ECOG PS of 1 after multiple treatment failures, fifth-line combination immunotherapy was initiated: paclitaxel, atezolizumab (PD-L1 inhibitor), and anlotinib. A PR was documented after two cycles (**Figure 1g**) and sustained for a PFS of 8 months until December 2024 when disease progression recurred (**Figure 1h**).

Sixth-line therapy with sacituzumab tirumotecan (TROP2-ADC) was discontinued after one due to severe oral mucositis. Restaging on February 24, 2025 confirmed disease progression, and a second bronchoscopic biopsy was obtained (designated the “post-immunotherapy/resistant specimen” [ID:25-801]). Tissue NGS revealed an EGFR 19del mutation (VAF 15.56%), EGFR exon 20 T790M missense mutation (VAF 7.78%), TP53 mutation (VAF 14.28%), and EGFR exon 8 splice site mutation. The patient succumbed to disease in August 2025; total OS was 51 months, including 16 months following fifth-line immunotherapy initiation (**Figure 2**).

**Figure 2:**
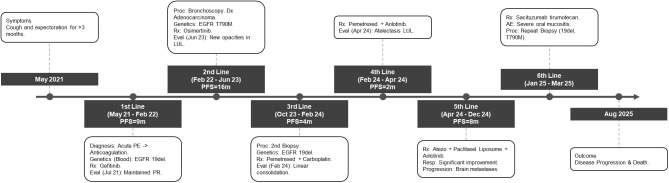
Timeline of clinical management and disease trajectory. Chronological summary of the patient’s treatment history, key response assessments, biopsy collection timepoints, and survival milestones from initial diagnosis (May 2021) through death (August 2025). Lines of therapy (first through sixth), PFS per line, biopsy identifiers (Specimen 23-2554: pre-immunotherapy; Specimen 25-801: post-immunotherapy progression), and OS endpoint are indicated. Total OS was 51 months; OS after fifth-line immunotherapy initiation was 16 months.

## 3 Materials and methods

To elucidate the immune mechanisms underlying the durable clinical response observed in this patient following resistance to standard TKI and chemotherapy, we performed the following analyses.

### 3.1 Specimen collection and study design

Two serial FFPE biopsy specimens were analyzed

- Pre-immunotherapy specimen (ID: 23-2554; collected June 28, 2023): Obtained after osimertinib resistance but before immunotherapy initiation; represents the “responsive” TIME state.
- Post-progression specimen (ID: 25-801; collected February 24, 2025): Obtained after failure of combination immunotherapy and ADC therapy; Represents the “resistant” TIME state.

### 3.2 Multiplex immunofluorescence staining

Dynamic quantitative assessment of immune cell infiltration and TLS was performed using a tyramide signal amplification multiplex immunofluorescence (TSA-mIF) approach. mIF staining was performed with a 7-color fluorescence immunohistochemistry kit (PhenoVision Bio Co., Ltd.) following the manufacturer’s instructions. Briefly, tissue sections were deparaffinized, rehydrated, and subjected to antigen retrieval in citrate buffer (pH 6.0). After washing, sections were incubated with H_2_O_2_ and blocking reagents for 10 min to quench endogenous peroxidase activity and minimize non-specific binding.

For PD-L1 detection, tissue sections were incubated with a rabbit monoclonal anti-PD-L1 antibody (clone E1L3N, Cell Signaling Technology, Danvers, MA, USA) for 30 min at room temperature. Bound primary antibody was detected using PVB anti-Rabbit/Mouse-HRP detection reagent, followed by PVB-690 for 10 min.

Sequential staining rounds for the remaining targets (CD3, CD20, CD68, pan-cytokeratin, PD-1, CD4, CD8, Ki-67, CD21, and CD23) were subsequently performed following the same TSA-mIF workflow. Finally, slides were counterstained with spectral 4′,6-diamidino-2-phenylindole (DAPI; PhenoVision Bio Co., Ltd.) and coverslipped.

### 3.3 Panel design

All antibodies were purchased from Cell Signaling Technology (Danvers, MA, USA).

- Panel 1 (Baseline immune microenvironment): CD3 (T cells), CD20 (B cells), CD68 (macrophages), panCK (tumor cells), PD-1, and PD-L1.
- Panel 2 (TLS maturity and proliferation): CD4 (helper T cells), CD8 (cytotoxic T cells), Ki-67 (proliferation), CD20 (B cells), CD21 (follicular dendritic cells, [FDCs]), CD23 (germinal center[GC] B cells/FDCs).

### 3.4 Image acquisition, data analyzed and TLS classification

Whole-slide mIF images were acquired using a PhenoImager HT system (Akoya Biosciences) at 400× magnification. All slides were scanned under identical acquisition settings to ensure consistency across samples.

#### 3.4.1 Region of interest selection

Regions of interest (ROIs) were selected through a standardized workflow. First, each stained slide was reviewed by a board-certified pathologist to identify tissue areas containing lymphocyte aggregates and putative tertiary lymphoid structures (TLSs). TLS regions were defined morphologically as organized lymphoid aggregates containing CD20-positive B cells within the tumor or stromal compartment.

All identifiable TLS-containing regions within each section were included for analysis. ROIs were manually annotated based on pathologist guidance and tissue morphology, ensuring that both intratumoral and stromal compartments containing TLS features were captured. Non-informative areas (e.g., tissue folds, necrosis, or artifacts) were excluded. The same ROI selection criteria were applied uniformly across all cases to minimize selection bias.

#### 3.4.2 Field of view and quantification strategy

Within each annotated ROI, all analyzable fields of view were included in the quantitative analysis. No random subsampling was applied. This approach ensured that the entire TLS-containing tissue area was evaluated rather than selected representative fields, thereby reducing sampling variability.

Quantitative metrics were calculated based on the total number of positive cells and their proportion relative to the total tissue area and the total TLS area (when applicable).

#### 3.4.3 Image analysis platform and phenotyping

Image analysis was performed using Visiopharm software (Oncotopix Discovery system, version 4.5.6.5; Hoersholm, Denmark). The analytical workflow included:

- Tissue segmentation (tumor vs. stromal regions when applicable)
- Cell segmentation using nuclear counterstaining
- Phenotype classification based on multiplex marker co-expression

Cell phenotypes were defined using predefined threshold-based algorithms developed within Visiopharm and validated during panel optimization. Identified phenotypes included, but were not limited to:

CD68/PD-L1+, CD3+/PD-L1+, CD20+/PD-L1+,

CD68/PD-1+, CD3+/PD-1+, CD20+/PD-1+,

Ki-67+/CD20+, CD23+/CD20+, and CD21+/CD20+.

TLS classification was performed based on marker expression patterns:

- TLSs containing CD21+ and CD23+ cells were classified as mature TLSs.
- TLSs lacking these features were classified as early-stage TLSs.

All analysis parameters (thresholds, segmentation rules, and phenotype definitions) were kept constant across all cases to ensure methodological consistency.

#### 3.4.4 Quality control and analytical consistency

To ensure reproducibility and minimize analytical variability:

- All slides were processed using the same staining protocol and imaging platform.
- Image acquisition parameters were standardized across samples.
- The same Visiopharm analysis pipeline and algorithm settings were applied to all cases.
- Pathologist-guided ROI selection was performed using predefined criteria.
- Data extraction was conducted in a uniform manner for all samples.

This standardized workflow was designed to reduce inter-sample variability and improve analytical rigor.

## 4 Results

### 4.1 Quantitative analysis of immune infiltration

Quantitative mIF analysis (**Figure 3**) revealed clear immune status differences between the two specimens (**Figure 4a**). In the pre-immunotherapy “responsive” specimen (23-2554), the CD3+ T-cell proportion was 3.85% compared with 1.69% in the post-progressing “resistant” specimen (25-801). CD20+ B-cell infiltration was markedly higher in the responsive specimens (4.82% vs. 0.72%). CD68+ macrophage proportions were comparable (1.17% vs. 1.13%) (**Figure 4b**).

**Figure 3:**
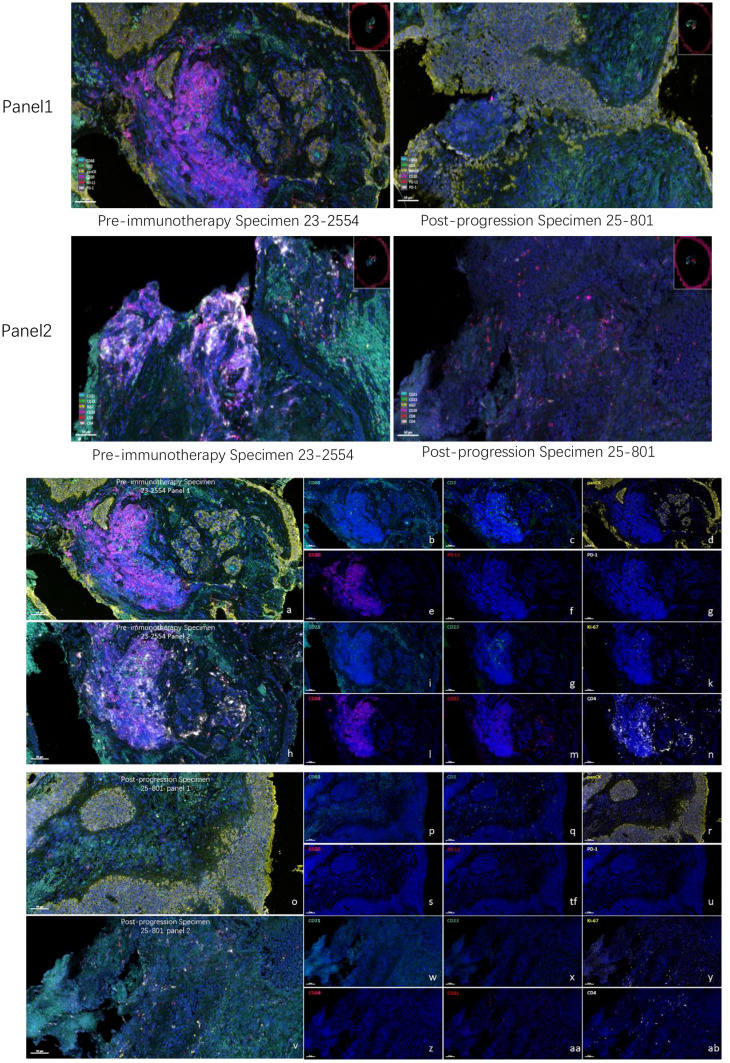
Representative multiplex immunofluorescence (mIF) images characterizing the tumor immune microenvironment (TIME) and tertiary lymphoid structure (TLS) maturity in matched tumor specimens collected prior to immunotherapy and at post-progression. **(a–g)** Panel 1 (baseline immune microenvironment) in the pre-immunotherapy specimen (23-2554): **(a)** merged composite image; **(b)** CD68; **(c)** CD3 (T cells); **(d)** pan-cytokeratin (panCK, tumor epithelial cells); **(e)** CD20 (B cells); **(f)** PD-L1; **(g)** PD-1 positive staining in the identical area. (h–n) Panel 2 (TLS maturity and proliferation) in the pre-immunotherapy specimen (23-2554): **(h)** merged composite image; **(i)** CD23 [germinal center B cells/follicular dendritic cells (FDCs)]; **(j)** CD21 (FDCs); **(k)** Ki-67 (proliferation marker); **(l)** CD20 (B cells); **(m)** CD8 (cytotoxic T cells); **(n)** CD4 (helper T cells). (o–u) Panel 1 in the post-progression specimen (25-801): **(o)** merged composite image; **(p)** CD68; **(q)** CD3; **(r)** panCK; **(s)** CD20; **(t)** PD-L1; **(u)** PD-1. (v–ab) Panel 2 in the post-progression specimen (25-801): **(v)** merged composite image; **(w)** CD23; **(x)** CD21; **(y)** Ki-67; **(z)** CD20; **(aa)** CD8; **(ab)** CD4. Scale bars, 50 µm. All antibodies were purchased from Cell Signaling Technology (Danvers, MA, USA).

**Figure 4:**
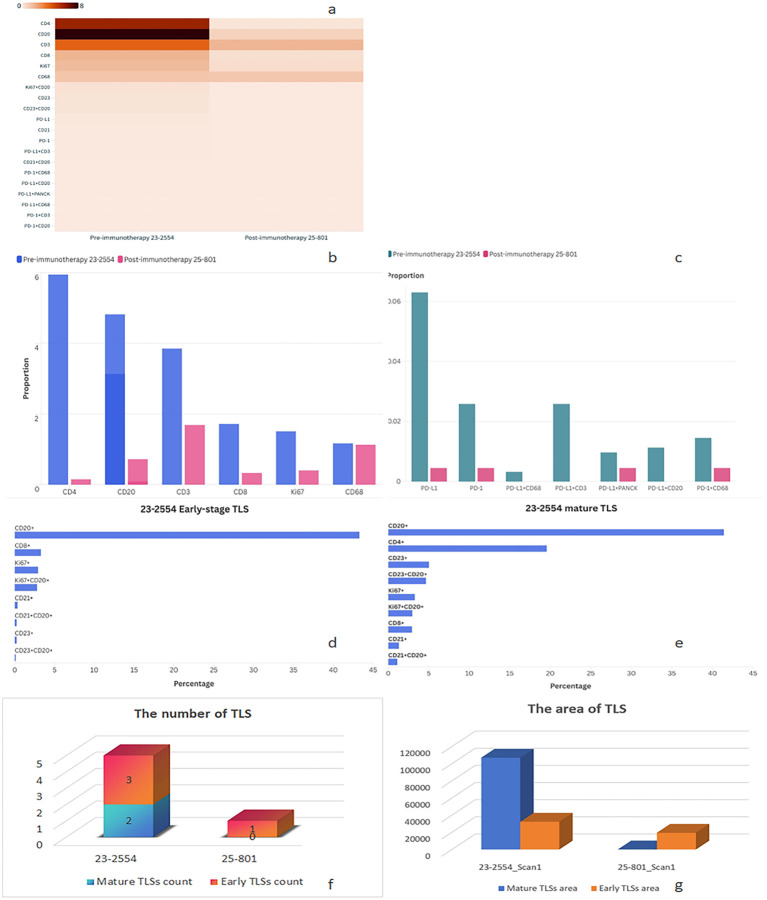
Quantitative multiplex immunofluorescence (mIF) analysis of the tumor immune microenvironment (TIME) and tertiary lymphoid structures (TLSs) in matched pre-immunotherapy (responsive, 23-2554) and post-progression (resistant, 25-801) specimens. **(a)** Heatmap visualization of immune cell proportions and marker co-expression patterns across the two specimens. Rows represent individual markers or marker combinations; color intensity indicates relative abundance (scale: 0–8). **(b)** Proportions of major immune infiltrates—CD4+ T cells, CD20+ B cells, CD3+ T cells, CD8+ T cells, Ki-67+ proliferating cells, and CD68+ macrophages—compared between the pre-immunotherapy (blue) and post-progression (pink) specimens. **(c)** Proportions of immune checkpoint–positive populations, including PD-L1+, PD-1+, and their co-expression with CD68, CD3, pan-cytokeratin (panCK), and CD20, in the pre-immunotherapy (teal) and post-progression (pink) specimens. **(d, e)** Cellular composition of early-stage TLSs and mature TLSs in 23-2554, showing the percentage of each phenotypic subset within the TLS region. **(f)** Quantification of TLS numbers. The pre-immunotherapy specimen (23-2554) harbored 2 mature TLSs and 3 early-stage TLSs, whereas the post-progression specimen (25-801) contained only 1 early-stage TLS and no mature TLSs. **(g)** Quantification of TLS areas. Comparison of mature TLS (blue) and early-stage TLS (orange) areas between the pre-immunotherapy (23-2554_Scan1) and post-progression (25-801_Scan1) specimens.

Regarding immune checkpoint expression, the overall PD-L1+ cell rate was 0.063% (responsive) vs 0.0045% (resistant). PD-L1+CD3+ cells (0.0258% vs. 0%) and PD-L1+CD20+ cells (0.0113% vs. 0%) were detectable only in the responsive specimens. PD-1+CD68+ expression was 0.0145% vs. 0.0045% (**Figure 4c**).

### 4.2 Evaluation of TLS maturity and proliferation

Intratumoral TLS analysis within the responsive specimen (23-2554) revealed markedly distinct cellular compositions between early-stage and mature TLSs (**Figures 4d, e**). In early-stage TLSs, CD20+ B cells constituted the dominant population (43.26%), with minimal contributions from CD8+ T cells (3.28%), Ki-67+ proliferating cells (2.92%), and follicular dendritic cell markers (CD21+ and CD23+, 0.36% and 0.24%, respectively) (**Figure 4d**). By contrast, mature TLSs exhibited a more diverse and organized immune architecture: CD20+ B cells remained predominant (41.39%), but CD4+ helper T cells were substantially enriched (19.54%). Moreover, mature TLSs contained higher proportions of CD23+ germinal center B cells (5.02%), CD23+CD20+ double-positive cells (4.65%), and Ki-67+ proliferating cells (3.26%), consistent with active germinal center formation (**Figure 4e**).

TLS enumeration further demonstrated a sharp decline in both number and maturity stage following disease progression. The pre-immunotherapy responsive specimen contained 2 mature TLSs and 3 early-stage TLSs, whereas the post-progression resistant specimen harbored only 1 early-stage TLS and no mature TLSs (**Figures 4f, g**).

## 5 Discussion

In this case, a patient who experienced failure of multiple lines of TKI and chemotherapy achieved an OS of 16 months following a PD-L1 inhibitor-based combination regimen, a clinically noteworthy outcome that prompted in-depth characterization of the post-resistance immune microenvironment.

Serial mIF analysis of biopsy specimens obtained pre-immunotherapy (Specimen 23-2554) and post-immunotherapy progression (Specimen 25-801) (**Figure 3**) revealed a paradox in the “responsive” specimen: despite low PD-1/PD-L1 expression (**Figure 4**)—a feature typically associated with poor ICI response (4)-- the TIME exhibited dense CD3+ T-cell and CD20+ B-cell infiltration, with comparatively low CD68+ macrophages infiltration (10, 11) (**Figure 4**).

Published evidence suggests that mature TLS are associated with ICI efficacy in NSCLC, independently of PD-L1 status and TMB (9, 12); however, these findings derive from larger observational cohorts and cannot be extrapolated to a single case. In the present report, the characterization of TLS as a potentially contributing feature is strictly observational. While this temporal co-occurrence is consistent with hypotheses regarding TLS-associated immune maintenance, it does not establish causality, and alternative explanations — including prior therapy effects, clonal evolution, and biopsy-site heterogeneity — cannot be excluded.

Despite inherent single-case limitations, this report highlights several directions for future investigation. Prospective studies evaluating TLS status in pre-treatment biopsies of EGFR TKI-resistant NSCLC patients could test the hypothesis that mature TLS, as defined by CD21+/CD23+ criteria, correlates with ICI response in this population. Standardization of TLS assessment — including consensus digital pathology workflows, multi-observer validation, and automated image analysis — will be critical for translating TLS characterization to clinical practice. Temporal profiling of the TIME across sequential treatment lines, ideally via serial biopsies or longitudinal liquid biopsy platforms, could help characterize TLS dynamics in relation to therapeutic interventions.

While this study provides quantitative characterization of TLS-associated immune phenotypes using multiplex immunofluorescence, several limitations should be acknowledged.

First, this analysis was performed on a limited number of samples, and therefore the quantitative findings should be interpreted as exploratory. Second, although multiple ROIs and all identifiable TLS-containing regions were analyzed within the available tissue section, spatial heterogeneity inherent to tumor immune microenvironments may not be fully captured by single-section analysis. Third, single-sample quantitative evaluation may not fully represent the dynamic or systemic immune landscape of the patient. Fourth, all specimen analyses were performed by a single pathologist in the absence of blinded review or consensus validation, and this limitation may lead to subjective assessment bias. In addition, individual drug efficacy cannot be distinguished under this triple-combination regimen. Preclinical data suggest paclitaxel-initiated immunogenic cell death and anlotinib-mediated TIME remodeling synergize to boost PD-L1 inhibitor responsiveness, partially explaining the observed clinical benefit (13–15).

Future studies including larger cohorts, multi-region sampling, dual-pathologist validation and independent validation datasets will be necessary to confirm these findings and improve generalizability.

## 6 Conclusion

These observations are exploratory and hypothesis-generating. The presence of mature TLS at the time of immunotherapy initiation may represent a feature of interest in EGFR-TKI-resistant NSCLC, but its role as a predictive biomarker cannot be established from a single case. Dynamic characterization of the TIME — and of TLS maturity status in particular, using standardized CD21/CD23-based criteria — merits investigation in prospective multicenter studies with adequate statistical power to define its clinical utility.

## Data Availability

The original contributions presented in the study are included in the article/supplementary material, further inquiries can be directed to the corresponding author/s.
